# When natural antibodies become pathogenic: autoantibodies targeted against G protein-coupled receptors in the pathogenesis of systemic sclerosis

**DOI:** 10.3389/fimmu.2023.1213804

**Published:** 2023-06-08

**Authors:** Reza Akbarzadeh, Antje Müller, Jens Y. Humrich, Gabriela Riemekasten

**Affiliations:** Department of Rheumatology and Clinical Immunology, University of Lübeck, Lübeck, Germany

**Keywords:** autoimmunity, inflammation, systemic sclerosis, autoantibodies, G protein-coupled receptor

## Abstract

Systemic sclerosis (SSc) is a chronic, multisystem connective tissue, and autoimmune disease with the highest case-specific mortality and complications among rheumatic diseases. It is characterized by complex and variable features such as autoimmunity and inflammation, vasculopathy, and fibrosis, which pose challenges in understanding the pathogenesis of the disease. Among the large variety of autoantibodies (Abs) present in the sera of patients suffering from SSc, functionally active Abs against G protein-coupled receptors (GPCRs), the most abundant integral membrane proteins, have drawn much attention over the last decades. These Abs play an essential role in regulating the immune system, and their functions are dysregulated in diverse pathological conditions. Emerging evidence indicates that functional Abs targeting GPCRs, such as angiotensin II type 1 receptor (AT1R) and the endothelin-1 type A receptor (ETAR), are altered in SSc. These Abs are part of a network with several GPCR Abs, such as those directed to the chemokine receptors or coagulative thrombin receptors. In this review, we summarize the effects of Abs against GPCRs in SSc pathologies. Extending the knowledge on pathophysiological roles of Abs against GPCRs could provide insights into a better understanding of GPCR contribution to SSc pathogenesis and therefore help in developing potential therapeutic strategies that intervene with pathological functions of these receptors.

## Introduction

**Figure 1:**
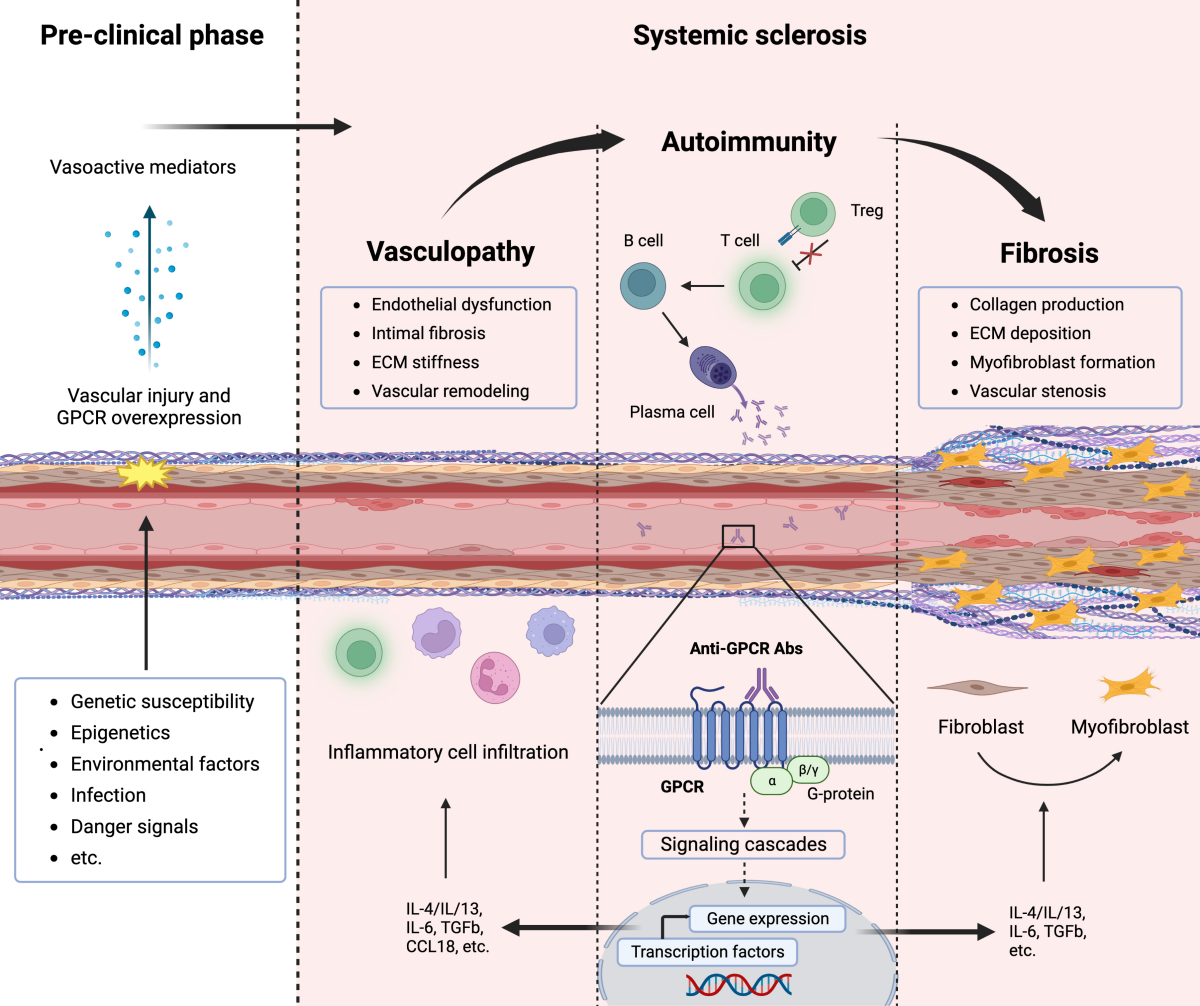
Pathogenesis of systemic sclerosis (SSc) and contribution of G-protein coupled receptors (GPCRs). The interplay between vasculopathy, autoimmunity, and fibrosis delineates the cardinal features of SSc pathogenesis. A microvascular injury is an early event in SSc that cause vascular pathologies such as vascular remodeling. Progressive vascular damage results in apoptosis, and dysregulation of the immune system (autoimmunity), where autoantibodies against various targets including GPCRs as well as proinflammatory cytokines are produced. Autoantibodies against GPCRs activate these receptors that amplify the inflammatory responses through their signaling pathway. Releasing mediators from smooth muscle cells and accumulating inflammatory cells cause severe vascular dysfunction (vasculopathy). Further endothelial damage and fibroblast conversion to the myofibroblast and extracellular matrix (ECM) deposition initiate the fibrotic process (fibrosis), which causes vessel stenosis and obliteration.

Systemic sclerosis (SSc) is a severe and chronic autoimmune disease of yet unknown etiology with high morbidity and disease-related mortality. Immune abnormalities, vasculopathy, and fibrosis are the cardinal hallmarks of SSc pathogenesis (1). Although the precise pathogenesis of SSc remains enigmatic, an interaction between genetic susceptibility and environmental factors, such as viruses or other infectious agents most likely play a significant role in the onset of the disease (2). The initiation phase usually involves microvascular injury with endothelial activation and vascular damage, which is followed by inflammation, Abs production directed against several antigens including GPCRs, and immune cell recruitment into the tissue, leading to vasculopathy and fibrosis (3, 4) (**Figure 1**). Progressive occlusive vasculopathy, the central feature of clinical manifestations in SSc, is present in nearly all SSc patients and starts from Raynaud’s phenomenon that extends during the disease process and can cause digital ulcers (DU) and pulmonary arterial hypertension (PAH) (5). The extreme obliteration of the renal vessels in SSc patients with scleroderma renal crisis (SRC) indicates that SRC is probably the most severe vascular complication in SSc (6). Besides vasculopathy, fibrosis is another hallmark feature of SSc and a variable extent of fibrosis has been found in the skin and lung, which is preceded by inflammation (1).

In SSc, some Abs target endothelial antigens and induce the production of pro-inflammatory cytokines, swarming of perivascular immune cells, and vascular remodeling and dysfunction (7). While natural Abs against GPCRs are identified in healthy individuals and are involved in physiological homeostasis through intracellular signaling pathways and coupling with heterotrimeric G proteins (8), dysregulation of these Abs mediates several immunopathological mechanisms that induce the development of autoimmune diseases such as SSc (9, 10). Pathogenic and functionally active Abs such as those against angiotensin 1 receptor (AT1R), endothelin A receptor (ETAR), C-X-C motif chemokine receptor 3 (CXCR3), and CXCR4 have been reported in the sera of SSc patients, regulating the spectrum of clinical manifestations (9, 11–14). Here, we sum up the current concept of involving Abs against GPCRs in the pathogenesis of SSc.

## Natural Abs against GPCRs: dual role in homeostasis and immunopathogenesis

Natural Abs against various GPCRs with agonistic or antagonistic activity are frequent in human serum, and these interactions contribute to the regulation of physiological processes, including immune responses (8). For a long time, it has been thought that Abs against GPCRs necessarily result in autoimmune diseases (15). Growing emphasis on the role of these Abs in controlling autoimmunity as well as their protective roles against the development of immune-mediated diseases such as psoriasis and type 1 diabetes, has led to a paradigm shift in determining the influence of GPCR Abs in regulating both physiological and pathophysiological processes (16). GPCRs, the typical chemokine receptors, are involved in host immune responses by functionally interacting with various physiological molecules such as growth factors and growth factor receptors that regulate the spatiotemporal control of effector cell dynamics and navigation of cell trafficking (17, 18). In this process, functional Abs against GPCRs could play a role in the maintenance of hemostasis by controlling immune cell functions such as migration or their stimulation in the secretion of cytokines and chemokines, which help in sustaining the physiological levels of these mediators in blood (7, 19). We recently demonstrated that there is a network of Abs against GPCRs in sera of healthy individuals that communicate with other Abs directed against growth factors and their respective receptors (8). For instance, Abs against ETRA from healthy donors modulate neutrophil migration directly through IgG-mediated chemotactic activity induced by the Fab fragment of IgG or indirectly via triggering interleukin-8 (IL-8) production by peripheral blood mononuclear cells (PBMCs) (8). Abs against GPCRs such as AT1R, ETAR, CXCR3, and CXCR4 can attract immune cells that express the corresponding receptors or be attracted by those cells, which is similar to the interaction of endogenous ligands to their receptors (8, 13, 19, 20). These effects seem to be important in immune cell homeostasis between blood and tissues, in which a balance between the serum levels of Abs against GPCRs and GPCR expression of immune or tissue-resident cells regulate cell migration towards the tissues and therefore prevent a systemic immune response to an acute local injury (21).

Besides the physiological roles, the levels of Abs against GPCRs are altered in pathological conditions such as SSc, and both increased and decreased concentrations correlate with the development or progress of immune-mediated disorders as well as disease specificity (8–10, 13, 22). Particularly in SSc, elevated levels of Abs against ATR1 and ETAR are found in approximately 85% of SSc patients, where the high concentrations of these two IgGs are associated with more aggressive disease and poor prognosis (9, 23). These correlations seem to be a consequence of cross-reactivities between the Abs against GPCRs. The contractile response of AT1R and ETAR to their natural ligands, i.e. angiotensin II and endothelin-1 is boosted by patient-derived IgG containing Abs against AT1R and ETAR, where considerable crosstalk between both receptors is noticed in driving autoimmune receptor hypersensitization (24). Moreover, the presence of Abs against CXCR3 and CXCR4 in SSc patients indicates a link between autoimmunity and interstitial lung disease, in which both Abs strongly correlate with each other and their concentrations can discriminate patients with stable or decreasing lung function (13). This may suggest that the levels of both Abs, as well as their correlations, provide significant information in understanding their potential contribution to the pathogenesis of SSc disease.

## Functional Abs against GPCRs in the pathogenesis of SSc

**Figure 2:**
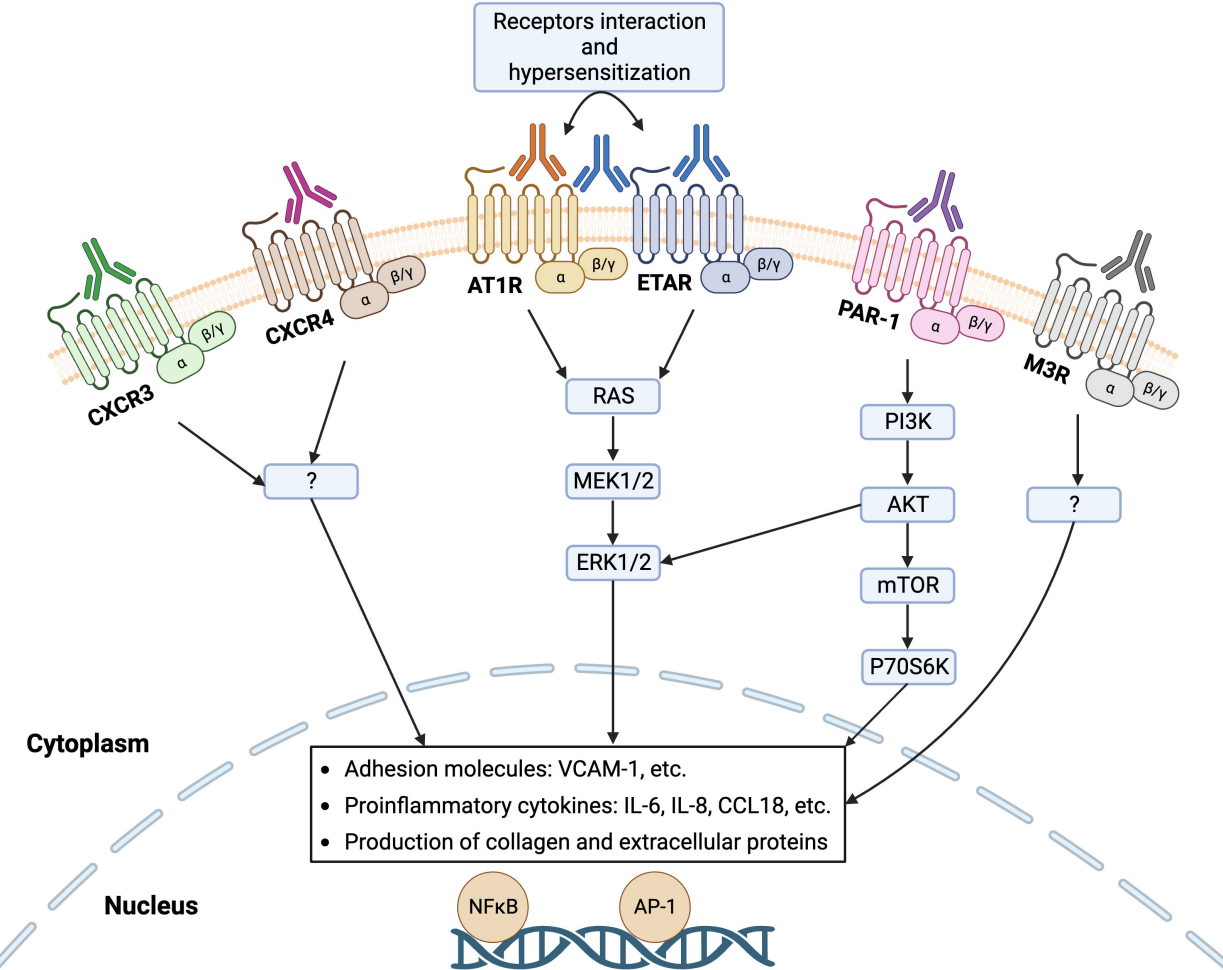
Functional autoantibodies against directed various GPCRs in systemic sclerosis (SSc). Autoantibodies against angiotensin II type 1 receptor (AT1R), endothelin-1 type A receptor (ETAR), C-X-C motif chemokine receptor 3 (CXCR3), CXCR4, protease-activated receptor-1 (PAR-1), and Muscarinic-3 Acetylcholine Receptor (M3R) have so far identified in SSc that can activate the respective receptors on endothelial cells and engage signaling pathway that regulates the transcription of several genes, such as increased gene expression of adhesion molecules, proinflammatory cytokines, and proteins of extracellular matrix (ECM). Interaction between the receptors (heterodimerization) such as AT1R and ETAR mediate receptor hypersensitization that may increase their sensitivity to the natural ligands.

GPCRs are the largest superfamily of integral membrane proteins that share a common structural architecture of seven transmembrane segments, connected by three extracellular and three intracellular amino acid loops, an extracellular N-terminus region, and an intracellular C-terminus (25). Upon activation by a ligand, GPCRs mediate a diverse array of intracellular signaling cascades through binding to a partner heterotrimeric G protein, which dissociates the G protein into α and βγ subunits (26). GPCRs are omnipresent on the surface of innate and adaptive immune cells such as leukocytes and lymphocytes as well as non-immune cells including endothelial cells and fibroblasts (27). Similar to the endogenous ligands of GPCRs, functional Abs can bind these receptors to promote or block intracellular signaling pathways, executing agonistic or antagonistic effects, respectively. In the last decade, a growing number of GPCRs have been noticed that are targeted by Abs in SSc disease such as functional Abs with agonistic effects on AT1R, ETAR, CXCR3, CXCR4, and protease-activated receptor-1 (PAR-1) as well as Abs with antagonistic effects on Muscarinic-3 Acetylcholine Receptor (M3R), where altered levels of these Abs, their cross-reactivity, and their synergistic effects influence the SSc pathogenesis (**Figure 2**). Here, we extensively summarize the evidence for the contribution of various Abs against selected GPCRs in the pathogenesis of SSc.

### Abs target vascular GPCRs: AT1R and ETAR

Angiotensin II (Ang II) and endothelin 1 (ET1) are identified to be elevated in blood and tissue samples of SSc patients (28, 29). AT1R and ETAR mainly contribute to the regulation of vascular function, production of extracellular matrix, the proliferation of vascular smooth muscle cells, and inflammatory responses (30). These GPCRs are activated by their natural ligands, i.e. Ang II and ET1, respectively, leading to actin polymerization and cytoskeletal remodeling in vascular cells that regulate blood pressure and immune cell migration (31–36). According to the evidence in SSc pathogenesis highlighting the contribution of Ang II and ET1 and their interaction with AT1R and ETAR via immunopathological pathways such as harmful vasoconstriction as well as pro-inflammatory and proliferative fibrosis, it is assumed that agonistic Abs targeting these vascular receptors could contribute to the pathogenesis of SSc (9). Mechanistic studies have so far confirmed the functional role of pathogenic Abs directed to AT1R and ETAR by targeting epitopes of the second extracellular loop of these GPCRs that maintain receptor activation (30). Functionally, Abs against AT1R/ETAR are stimulatory or agonistic to their endogenous ligands (10). Following the binding of these Abs to their respective GPCRs, pro-inflammatory and pro-coagulatory processes are activated by kinase signaling pathways such as p38-mitogen-activated protein kinase (MAPK), tyrosine-protein kinase Src, protein kinase G, and extracellular signal-regulated kinases (ERK) 1/2 as well as several transcription factors including activator protein 1 (AP-1) and nuclear factor-kB (NF-kB), and transforming growth factor-beta (TGFβ) (9, 19, 37–41). Moreover, agonistic activation of AT1R on the surface of immune cells, such as neutrophils, monocytes, B cells, and T lymphocytes induces pro-inflammatory gene expression, including IFN-γ, TNF-α, IL-1, IL-6, IL-8, and IL-17 (42–44). Chemokines including monocyte chemoattractant protein-1 (MCP-1) and cytokine-induced neutrophil chemoattractant-1 (CINC-1) are the major elements in prompting immune cell swarms into the tissues, including skin, lungs, and kidney (45–47). Here, activation of these receptors regulates immune cell trafficking including neutrophils and monocytes via chemotactic mechanisms and the production of various cytokines and altered expression of adhesion molecules (37, 48–53). Abs against AT1R/ETAR promote the expression of the vascular cell adhesion protein 1 (VCAM1) and the release of IL-8 cytokine or CC chemokine ligand 18 (CCL18) that causes increased recruitment of inflammatory immune cells like neutrophils into the skin of SSc patients (7, 19). CCL18, a potent chemoattractant for lymphocytes, is significantly expressed in patients suffering from chronic inflammatory diseases and altered levels of this chemokine have been noticed as a prognosis element for lung disease progression and mortality in SSc patients (19, 20, 54).

### Abs target chemoattractant GPCRs: CXCR3 and CXCR4

Chemokine GPCRs, such as CXCR3 and CXCR4 together with their respective ligands provide a complex that mediates several cell functions in physiological and pathological processes, leading to the regulation of cell migration (55). In SSc, inflammation precedes or is accompanied by fibrosis, where leukocyte accumulation, endothelium activation, fibroblast proliferation and differentiation as well as production of excessive collagen and other extracellular matrix proteins are the hallmarks of inflammatory fibrosis (3, 56). For instance, interstitial lung disease, a major cause of lung fibrosis, is one of the key features of SSc pathogenesis which is associated with mortality due to the activation and swarming of leukocytes and lymphocytes at the site of inflammation (57). While elevated expression of CXCR4 has been reported in the skin of SSc patients, variable (increased, reduced, or unchanged) presence of CXCR3-expressing immune cells such as T lymphocytes is seen in SSc (58–62). CXCR3 is extensively expressed on effector T cells that are activated by interferon gamma-inducible ligands at inflamed tissues and play a significant role in T cell trafficking and function (63). On the other hand, CXCR4 is the cardinal receptor for CXCL12 (also called stromal-derived factor 1, SDF-1), which form a network in attracting neutrophils and lymphocytes (64). Functional Abs against CXCR3 have been observed as modulators of the receptor with potential agonistic activity (8, 13, 65). Altered levels of Abs against CXCR3 and/or CXCR4 have been observed in SSc patients and the levels of these two Abs correlate with each other (8). For instance, higher levels of CXCR3 and CXCR4 Abs have been observed in the sera of SSc patients compared to the healthy controls, where higher levels of CXCR3 in patients with interstitial lung disease correlate with worse lung function (13). Noteworthy, the concentrations of Abs against CXCR3 and CXCR4 are different between SSc subgroups. Patients with deterioration of lung function had lower CXCR3/4 Abs compared to those with stable disease (13). This reduction in the levels of CXCR3/4 Abs with disease progression suggests a promising predictive value in lung disease severity of SSc. Nevertheless, the mechanism of action, signaling pathways, and functional activity of these Abs in SSc are still unknown.

### Abs target coagulation-related GPCRs: PAR-1

PAR-1, a GPCR expressed on various cell types such as endothelial cells and smooth muscle cells, is essential for the regulation of endothelial barrier function and pro-inflammatory cytokine production, hence plays a significant role in the interplay between inflammation and coagulation in various inflammatory conditions, including autoimmune diseases (66). Imbalance in the coagulative/fibrinolytic cascade is obvious in the pathogenesis of SSc, where fibrinolysis impairment and activation of the coagulation results in endothelial dysfunction (67). Moreover, microvascular thrombosis, platelet aggregation, and increased fibrin deposition are frequently observed in SSc (68). Compelling evidence has recently emerged that Abs against PAR-1 are present in SSc patients with SRC, a life-threatening complication of SSc, which agonistically induce PAR-1 activation through signaling pathways involved in the production of pro-inflammatory factors such as IL-6 (6). It has been reported that microvascular endothelial cells exposed to SSc-derived Abs mediate PAR-1 activation that engages a signaling cascade including ERK1/2-dependent activation of PI3K/AKT/mTOR/p70S6K and AP-1/c-FOS to activate the IL-6 promoter, leading to elevated secretion of IL-6 in a time- and dose-dependent manner (6). Recently, it was noted that functional Abs against PAR-1 predispose the activation of the coagulation system in coronavirus disease (COVID)-19, a disease that shares common pathologic alterations and similar pathways with SSc such as endothelial dysfunction, vasculopathy, coagulation, fibrinolysis, and increased D-dimer levels (69). Blockade of the endothelial PAR-1 or c-FOS/AP-1 silencing or SSc therapy by anti-IL-6 receptor antibody tocilizumab provides further evidence of involving Abs against the PAR-1 in the pathomechanism of SSc (6, 70).

### Abs target organ-specific GPCRs: M3R

Almost all SSc patients suffer from dysfunction of the gastrointestinal tract, which its severity correlates with high mortality (71). Intestinal dysmotility, a phenomenon caused by progressive fibrosis and increased collagen deposition in the organs, accounts for the most gastrointestinal manifestations in patients with SSc (72). Muscarinic acetylcholine receptors, consisting of five members M1 to M5, are GPCRs that are in charge of a vast number of mechanisms that contribute to the autoimmune diseases (73). In the gastrointestinal system, acetylcholine is released from cholinergic nerve terminals that regulate gastrointestinal motility through the M3R (74). Abs blocking M3R could potentially function as antagonists for this receptor that inhibit excitatory enteric neurotransmission causing dysmotility. It has been hypothesized that the presence of Abs against M3R could be involved in the pathogenesis of gastrointestinal dysmotility in SSc. Following this hypothesis, elevated levels of anti-myenteric neuronal Abs were found in the sera of SSc patients with gastrointestinal indications (75). Indeed, elevated levels of M3R Abs have been found in the serum of SSc patients that inhibit cholinergic neurotransmission and hence are associated with the development of gastrointestinal dysmotility (76–80). Further evidence indicates that Abs against M3R contribute to a consecutive development of dysmotility in SSc, which begins with neuropathy due to the blocking of cholinergic neurotransmission and is followed by myopathy due to the inhibition of acetylcholine action at the smooth muscle cells (81). Since intravenous immunoglobulin (IVIG) competes with pathogenic SSc Abs (80), it could be of interest that the replacement of Abs against M3R with IVIG impairs the binding of these IgGs to the receptors. In this regard, *ex vivo* studies have shown that IVIG improves the SSc gastrointestinal symptoms by neutralizing Abs against M3R (80, 81). More recently, a case report study described the IVIG therapy of two SSc patients with circulating M3R Abs and severe swallowing impairment, which improved after treatment with IVIG (82).

## Abs-mediated GPCR pathways in SSc: from *in vitro* studies to animal models

The contribution of Abs against GPCRs in the pathogenesis of SSc has been confirmed by both *in vitro* and *in vivo* studies, linking the major features of SSc pathogenesis, i.e. immune dysregulation, fibrosis, and vasculopathy. As shown by *in vitro* investigations, excessive collagen production by skin fibroblasts or increased release of reactive oxygen species (ROS) by neutrophils is induced by Abs against AT1R and ETAR, which are involved in mechanisms that contribute to progressive fibrosis of the skin and lungs as well as vasculopathy, resulting in organ dysfunction and failure (7, 83). Besides the *in vitro* experiments, animal models provide excellent opportunities to study the pathophysiological contribution of Abs against GPCR in disease development. In an *in vivo* model, passive transfer of SSc patient-derived Abs directed against AT1R and ETAR into mice induces similar disease features that are seen in patients with SSc, such as interstitial lung disease and obliterative vasculopathy (7, 23). In line, a humanized mouse model of SSc has been introduced by transferring PBMCs derived from SSc patients as well as healthy individuals into immunodeficient mice (84). In contrast to the controls, PBMC-recipient mice from SSc patients exhibit systemic inflammation and cellular infiltrates in several organs, such as lungs, muscles, and kidneys. Moreover, these mice display increased levels of circulating human Abs against AT1R in their sera. In another humanized animal model of SSc, human PBMCs from SSc patients were transferred into NOD.Cg-Prkdcscid Il2rgtm1Wjl/SzJ (NSG) mice, which are extremely immunodeficient mice and suitable for being humanized by engraftment of human cells (85). This PBMC-transfer model indicated severe inflammation, vasculopathy, and fibrosis in the skin and lungs as well as elevated expression of GPCRs on infiltrated T helper cells, such as CXCR3.

More recently, we immunized C57BL/6J mice as well as mice deficient for B and T cells with membrane-embedded human AT1R (86). While immunodeficient mice are protected, immunized immunocompetent mice developed functional AT1R IgG as well as SSc-like inflammatory manifestations in the perivascular organs, including skin and lungs. In this study, further generation of a monoclonal anti-AT1R antibody by using the hybridoma technique and transferring it into the wild-type mice induces lung and skin inflammation, but AT1R-deficient mice remain unaffected (86). These observations are in agreement with clinical data that SSc disease correlate with altered levels of Abs against AT1R (9). However, since these Abs are also frequent in healthy individuals, they may pave the way for susceptibility to other severe organ inflammation, such as the involvement of skin and lungs in patients with COVID-19 (8).

## Targeting GPCRs and respective Abs: a potential therapeutic approach

SSc treatment is challenging, as it is a clinically heterogeneous disorder and its pathomechanism is not yet fully understood. Although GPCRs and corresponding Abs have been a major target for drug discovery in the last decades (87–89), the clinical applications of anti-GPCR agents in systemic autoimmune diseases are confined, due to the activation of multiple pathways by GPCRs that can restrict the drug efficacy or induce undesirable effects. So far approved anti-GPCR drugs such as endothelin receptor blockers that block AT1R and ETAR are currently used as a treatment strategy, which is mainly based on treating clinical complications of SSc such as vasculopathy, PAH, and DU (90–92). A recent randomized placebo-controlled phase II study on the endothelin-1 receptor blocker Zibotentan indicates the capacity of ETAR blockade to improve long-term renal function (93).

In recent years, many attempts have tried to improve the therapeutical strategies that can selectively and specifically target Abs or their receptors, which could provide new insights into the treatment of autoimmune diseases including SSc. Neutralization or elimination of pathogenic Abs has been successfully examined in several Abs-mediated pathologies, such as autoimmune bullous diseases (94). Despite being a promising strategy, conventional immunoadsorption can only temporarily diminish the Abs levels and should be usually complemented by adjuvant therapy, including immunosuppression to suppress the later production of Abs. This issue is also obvious in treatments of SSc and the levels of Abs against GPCRs such as AT1R and ETAR are quickly elevated when immunoadsorption is interrupted (12). To overcome this problem, an improved immunoadsorption approach has recently been applied by using nucleic acid aptamers or so-called ‘chemical antibodies’, which are single-stranded RNA or DNA oligonucleotide sequences that specifically target proteins bind to a molecule in its native conformation with a high affinity. Aptamer BC007, a thrombin inhibitor molecule, has been successfully used to neutralize functional Abs against GPCRs (95). Given the beneficial characteristics of aptamers in the neutralization of Abs, this approach could account for a promising potential therapeutic strategy in the treatment of SSc.

Another therapeutic option for the improvement of disease manifestations in SSc is autologous hematopoietic stem cell transplantation (AHSCT). Since its introduction in 1997, it has been used as an effective therapy for patients suffering from severe and rapidly progressive SSc refractory to immunosuppressive treatment (96–99). In this method, autoreactive autoantibody-producing cells such as B and T lymphocytes are eliminated by high-dose immunosuppressive drugs, followed by transplantation of hematopoietic stem cells, which is believed to exert an “immune reset”, leading to the restoration of dysregulated immune responses (100). However, the effect of this scenario on functional Abs against GPCRs is not yet well investigated. A recent study has shown that the reactivity and the levels of functional AT1R Abs are not touched by this therapy (101). Moreover, despite the advantages of the AHSCT, potential challenges such as providing an optimal therapeutic regimen, limitations in the availability of biomarkers that help in selecting patients who may benefit from transplantation, and late-onset adverse events remain to be determined (102).

Over the last two decades, potential therapeutic approaches have been introduced that are based on mechanisms beyond the canonical agonistic or antagonistic function of GPCRs and rely on selective effects of ligands activating specific G proteins or skewing signaling towards other pathways such as β-arrestin (103, 104). This concept is known as “biased” or “functional selectivity” ligand signaling, in which specific GPCR ligands preferentially promote signal transduction through one pathway (β-arrestin) over another (G-protein) and offers a new class of molecules intervening with the structural pathways of GPCRs and might improve efficacy and minimize the adverse effects of treatments. Receptor-ligand experiments indicate that G-protein and β-arrestin signaling are not necessarily activated unbiasedly by the ligands that bind to GPCRs, and agonist- or antagonist-biased ligands can alter the conformation of the receptors and activate the β-arrestin independent of G-protein (105). Hence, biased ligands can suppress the pathological effects of Abs throughout the GPCR signaling pathway by preferentially inducing the activation of β-arrestin, rather than G-protein. Since the existence of an on-off switch in this approach leads to the inactivation of G-protein signaling by uncoupling these proteins from GPCRs via the β-arrestin pathway, the biased ligand mechanism has been focused for establishing new therapeutical drugs to inhibit pathological effects of Ang II-mediated vasoconstriction through AT1R (106–108). Notably, the β-arrestin-biased AT1R ligand namely TRV023 has functional properties in cardioprotection distinctly from the traditional AT1R blocker, Losartan (109). Given the signaling of AT1R Abs in the pathogenesis of SSc, designing novel treatments that target the β-arrestin-biased AT1R signaling pathway could be a promising approach in the treatment of SSc. Several studies using another β-arrestin-biased AT1R agonist TRV027 could provide promising initial data in different animal models with heart complications (106, 110). Very recently, a β-arrestin-biased AT1R agonist TRV027 could prevent aortic aneurysm and respective mortality via distinct mechanisms than the AT1R blocker, Olmesartan (111). However, the TRV027 peptide failed to support this promising hypothesis in a clinical trial (107). This failure could be due to the functional differences between two distinct β-arrestin proteins in cardiac signaling, i.e. β-arrestin-1 and -2, which they are cardio-toxic and cardio-protective proteins, respectively (112). Given the higher expression of β-arrestin-1 in human cardiomyocytes (113), this pathway could be activated in the patients rather than β-arrestin-2, and thereby no favorable clinical outcomes have been observed. Considering these challenges, activation of the β-arrestin pathway by biased ligands does not necessarily provide desirable consequences in therapy, and designing GPCR-biased ligands that preferentially activate protective pathways might be helpful.

A further therapeutical approach for SSc has recently been introduced that pharmacologically blocks fibrosis through lysophospholipids (LPs) signaling that exerts their signal transduction through GPCRs. Lysophosphatidic acid (LPA), a bioactive lipid mediator present in all eukaryotic tissues, is generated from membrane phospholipids via different enzymatic pathways and drives diverse physiological and pathophysiological effects via its specific GPCR, namely LPA receptor (LPA1). LPA is highly generated at the sites of inflammation or cell injury and its signaling is associated with skin and pulmonary fibrosis, suggesting a potential therapeutic target in fibrotic diseases (114, 115). Recent evidence from *in vitro* studies or animal models as well as pre-clinical observations has proved the contribution of LPA to the pathogenesis of SSc or organ fibrosis, suggesting that LPA receptor (LPA1) antagonists may be effective in the treatment of SSc (116–119). The therapeutic potential of a new orally active antagonist of the LPA1 receptor, SAR100842, has been recently examined in SSc disease with using of dermal fibroblasts derived from patients and an animal model of skin fibrosis. This study demonstrates that SAR100842 has anti‐fibrotic effects through LPA1 receptor blockade, which is mediated by the inhibition of the Wnt signaling pathway in SSc fibroblasts as well as in the tight skin 1 (Tsk1) mice, a mouse model of skin fibrosis (120). Furthermore, the results of a double-blind, randomized, placebo-controlled study performed in patients with diffuse cutaneous SSc treated with an antagonist of LPA1, SAR100842, indicated that the therapy was well-tolerated and LPA-related genes, as well as Rodnan skin thickness score (MRSS), reduced, but these differences remained non-significant (121). Therefore, a larger clinical trial would need to evaluate these findings.

## Prospectives

Destructive pathologies and heterogeneity of SSc pose serious challenges in the development of curative therapeutic options. Recent advances in research focusing on the involvement of functional Abs against GPCRs that contribute to the pathological induction of intracellular signaling have expanded our knowledge in better understanding the mechanisms that contribute to SSc pathogenesis. Altered levels of Abs against GPCRs as well as their interplay influence the development and progression of SSc disease. Despite extensive efforts and scientific progress in understanding the function of Abs against GPCRs, it remains unclear how these IgGs regulate immune cell production, maturation, or migration. Moreover, it needs to be further determined the mechanisms behind the differential recruitment of immune cells such as leukocytes or lymphocytes to specific organs such as lungs and skin as well as the presence of fibrosis in one organ but missing in others, which could be an indication of tissue-specific nature of Abs against GPCRs. Due to their implications in the regulation of homeostasis and pathogenesis of autoimmune diseases including SSc, uncovering the pathophysiology of Abs directed against GPCRs might help design potential new therapeutical strategies.

## Author contributions

RA and GR contributed to the conception and design of the review. RA wrote the review and RA, AM, JYH, and GR reviewed and revised the final version. All authors have read and approved the submitted version.
